# Supplementary material for: The Ghrelin Analog GHRP-6, Delivered Through Aquafeeds, Modulates the Endocrine and Immune Responses of Sparus aurata Following IFA Treatment
Source: Biology (Basel). 2025 Jul 25;14(8):941. doi: 10.3390/biology14080941 (PMC12383794; doi:10.3390/biology14080941)
Supplement: Supplementary file 1 [file biology-14-00941-s001.zip › Figure S1.pdf]

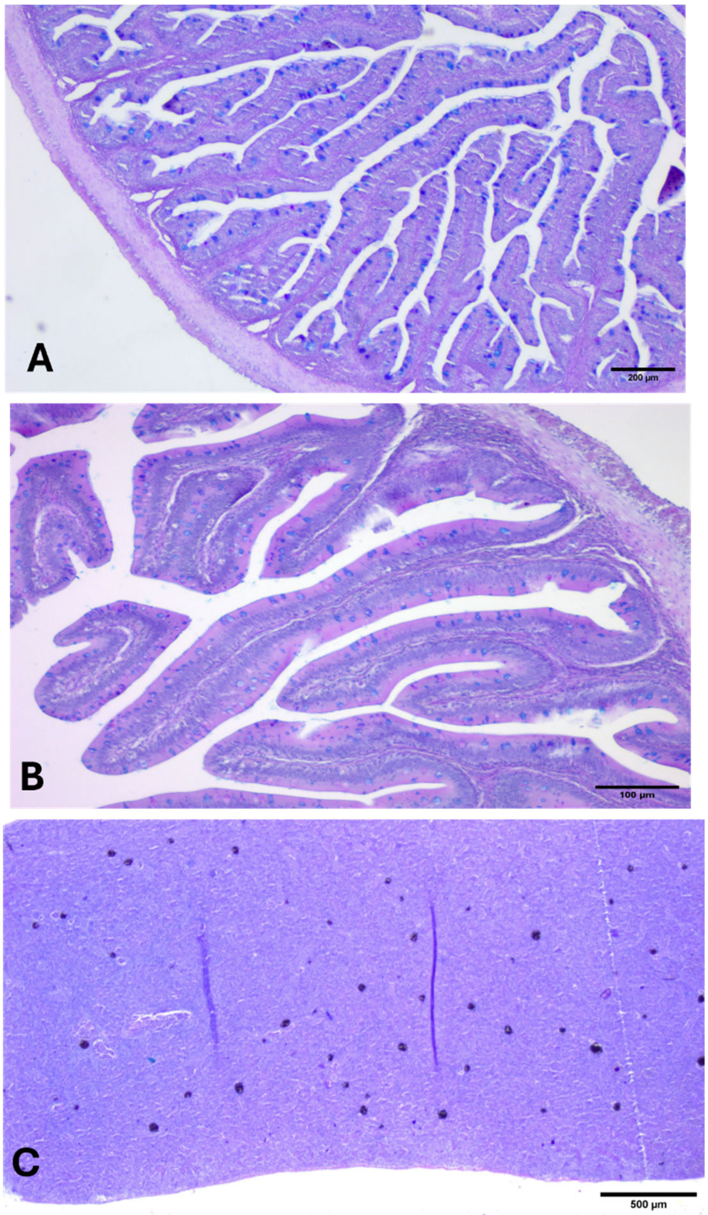

Figure S1. Photomicrograph of intestine and spleen of S. aurata from control experimental group. A. Normal histology of anterior intestine (H-E/AA 2.5/PAS). B. Normal histology of posterior intestine (H-E/AA 2.5/PAS). C. Normal histology of spleen (H-E).
