# Supplementary material for: The Ghrelin Analog GHRP-6, Delivered Through Aquafeeds, Modulates the Endocrine and Immune Responses of Sparus aurata Following IFA Treatment
Source: Biology (Basel). 2025 Jul 25;14(8):941. doi: 10.3390/biology14080941 (PMC12383794; doi:10.3390/biology14080941)
Supplement: Supplementary file 1 [file biology-14-00941-s001.zip › Table S1.pdf]

Table S1. The sequence of the primers used in qPCR. AI: anterior intestine; PI: posterior intestine.

Gene	Name	Sequence 5'-3'	Accession number	Amplicon size (bp)	R^2 and E Spleen	R^2 and E Head kidney	R^2 and E AI	R^2 and E PI
actb	saQactb-gF1	AGCCAACAGGGAGAAGATGA	XM_030406939.1	100	R^2 = 1.000<br>E = 99.9	R^2 = 0.998<br>E = 106.7	R^2 = 0.999<br>E = 98.7	R^2 = 0.997<br>E = 103.6
	saQactb-gR1	ACCAGAGGCATACAGGGACA						
ef1a	saQeef1a-gF1	GATGGCACGGTGACAACAT	AF184170.1	200	R^2 = 0.998<br>E = 98.3	R^2 = 0.998<br>E = 104.0	R^2 = 0.995<br>E = 100.4	R^2 = 0.998<br>E = 99.2
	saQeef1a-gR1	AGTTCCAATACCGCCGATTT						
**IMMUNOLOGICAL/INFLAMMATORY STATUS**								
il8	saQil8-gF1	ACTGCGAGGAGACCGAGAT	JX976619.1	187	R^2 = 0.992<br>E = 108.0		R^2 = 0.996<br>E = 99.4	R^2 = 0.999<br>E = 103.2
	saQil8-gR1	TTTGGTTGTCTTTGGTCGAA						
il10	saQil10-gF1	CGGAGGACACCAGGAACTTA	XM_030418889.1	199	R^2 = 0.997<br>E = 105.2		R^2 = 0.986<br>E = 92.8	
	saQil10-gR1	TTGAACAGCAGATCCAGCTC						
il15	saQil15-gF1	CATGTTGGAGTTGATGATGGTC	JX976625.1	191			R^2 = 0.998<br>E = 99.9	R^2 = 0.99<br>E = 90.8
	saQil15-gR1	CCCGTTCAGTCATTTCTTCC						
il34	saQil34-gF1	AGCATAGAAGCACAGCAGGA	XM_030427145.1	108	R^2 = 0.997<br>E = 97.8		R^2 = 0.995<br>E = 107.1	R^2 = 0.996<br>E = 92.9
	saQil34-gR1	GGTTGACATCGCTCCACTTT						
casp1	saQcasp1-gF1	GACTCACTCATCCCTGTCAAAG	XM_030438153.1	170	R^2 = 0.994<br>E = 99.0	R^2 = 1.000<br>E = 105.2		
	saQcasp1-gR1	TTCTCTTCGTCCTTCTCAGC						
lgals1	saQlgals1-gF1	GGCAGACCCTGACCATTG	KF862003.1	194	R^2 = 0.997<br>E = 109.6	R^2 = 0.999<br>E = 93.9	R^2 = 0.998<br>E = 99.1	R^2 = 0.994<br>E = 104.4
	saQlgals1-gR1	GGGAAAGCCTCCATCACG						
lgals8	saQlgals8-gF1	GTGCATCTCTGGACAGGTCA	KF862004.1	198		R^2 = 0.998<br>E = 106.9	R^2 = 0.996<br>E = 94.0	R^2 = 0.997<br>E = 94.0
	saQlgals8-gR1	TCTCCCTTGATGGCGATG						
ccr3	saQccr3-gF1	TGCAAACCTGGAATCAGTCA	XM_030401704.1	127			R^2 = 0.998<br>E = 103.7	R^2 = 0.998<br>E = 106.6
	saQccr3-gR1	GAGCACGTAACTCGGGTCAG						
ccr9	saQccr9-gF1	CCAGACAAAGGCAACAGAGC	XM_030397691.1	171			R^2 = 0.998<br>E = 105.0	R^2 = 0.989<br>E = 100.1
	saQccr9-gR1	TGCACATGACTGCGTTATCA						
mx1	saQmx1-gF1	ATCTCTGCCTCAACTGGAAA	FJ490556.1	148	R^2 = 0.989<br>E = 100.0		R^2 = 0.999<br>E = 103.4	R^2 = 0.996<br>E = 96.8
	saQmx1-gR1	GGCATCCTGAGTGAAAGCTG						
mx2	saQmx2-gF2	TGGCCCCGTACATCTTAAAA	FJ490555.1	182	R^2 = 0.999<br>E = 100.7	R^2 = 0.996<br>E = 102.9	R^2 = 0.990<br>E = 103.6	R^2 = 0.999<br>E = 100.6
	saQmx2-gR2	ACTCCTGCAACATCTGGTAGC						
mx3	saQmx3-gF1	AGAGATATGGCAATGGACCC	FJ652200.1	162				

	saQmx3-gR1	CAAAGTCGCTTCGGAGTGTA						
ighm	saQighm-F2	CCAAGGAGAAGTGGAACACA	XM_030408618.1	164	R^2 = 0.98<br>E = 103.3		R^2 = 0.992<br>E = 106.7	
	saQighm-R2	ACAGAACACGCCAGCACATA						
**MUCUS PRODUCTION AND GOBLET CELL DIFFERENTIATION**								
muc2	saQmuc2-gF1	TCACTGGAGAAACCACACCA	JQ277710.1	182			R^2 = 0.994<br>E = 107.8	R^2 = 0.995<br>E = 104.6
	saQmuc2-gR1	TGCCAGACTCTTGGTTGAAG						
muc3b	saQmuc3b-gF1	AAAGAGATACATGGGTGTTTGG	XM_030418634.1	200				R^2 = 0.993<br>E = 103.2
	saQmuc3b-gR1	AAACTTCTGGATGATTTGTTGC						
klf4	saQklf4-gF1	CTACGCCTCCTATCCTCAGC	XM_030435936.1	95			R^2 = 0.990<br>E = 107.1	R^2 = 0.987<br>E = 103.9
	saQklf4-gR1	GTTTGGGCTTTGGTTCCTC						
