# Supplementary material for: The Ghrelin Analog GHRP-6, Delivered Through Aquafeeds, Modulates the Endocrine and Immune Responses of Sparus aurata Following IFA Treatment
Source: Biology (Basel). 2025 Jul 25;14(8):941. doi: 10.3390/biology14080941 (PMC12383794; doi:10.3390/biology14080941)
Supplement: Supplementary file 1 [file biology-14-00941-s001.zip › Table S2.pdf]

Table S2. Statistical information obtained from the Two-way ANOVA test, and the multiple comparisons test carried out for gene expression level of juvenile gilthead sea breams (Sparus aurata) fed with a commercial (C) diet or supplemented with 500 µg GHRP-6/kg aquafeed (D) for 97 days, injected with 100 µL/g fish of saline solution (Sal) or Incomplete Freund's adjuvants (IFA) and sampled after 72 h post-injection.

**Genes**	*p*-diet	*p*-IFA	*p*-interaction
**Intestine anterior**			
il10	<0.001	0.740	0.913
il15	0.675	0.451	<0.001
il34	0.363	0.062	0.006
mx1	0.014	0.050	0.013
lgals1	<0.001	0.971	0.515
ccr9	0.215	0.181	0.009
**Intestine posterior**			
mx1	0.207	0.669	0.015
ccr3	0.813	0.002	0.923
muc2	0.002	0.092	0.075
muc3b	0.001	0.125	0.383
**Spleen**			
il18	0.015	<0.000	0.006
il10	0.085	0.002	0.093
il34	0.331	0.733	0.021
mx1	0.691	0.159	0.000
mx2	0.057	0.056	0.016
lgals1	0.010	0.260	0.738
ighm	0.259	0.116	0.022
**Head kidney**			
il15	0.290	0.454	0.001
